# Biodegradation of di-*n*-butyl phthalate by bacterial consortium LV-1 enriched from river sludge

**DOI:** 10.1371/journal.pone.0178213

**Published:** 2017-05-25

**Authors:** Yangyang Wang, Fangfang Li, Xinling Ruan, Jian Song, Lv Lv, Liyuan Chai, Zhihui Yang, Lin Luo

**Affiliations:** 1 School of Resources and Environment, Hunan Agricultural University, Changsha, China; 2 Key Research Institute of Yellow River Civilization and Sustainable Development & Collaborative Innovation Center on Yellow River Civilization of Henan Province, Henan University, Kaifeng, China; 3 Institute of Natural Resources and Environment, Henan University, Kaifeng, China; 4 School of Metallurgical & Environment, Central South University, Changsha, China; Universita degli Studi di Milano-Bicocca, ITALY

## Abstract

A stable bacterial consortium (LV-1) capable of degrading di-*n*-butyl phthalate (DBP) was enriched from river sludge. Community analysis revealed that the main families of LV-1 are *Brucellaceae* (62.78%) and *Sinobacteraceae* (14.83%), and the main genera of LV-1 are *Brucella* spp. (62.78%) and *Sinobacter* spp. (14.83%). The optimal pH and temperature for LV-1 to degrade DBP were pH 6.0 and 30°C, respectively. Inoculum size influenced the degradation ratio when the incubation time was < 24 h. The initial concentration of DBP also influenced the degradation rates of DBP by LV-1, and the degradation rates ranged from 69.0–775.0 mg/l/d in the first 24 h. Degradation of DBP was best fitted by first-order kinetics when the initial concentration was < 300 mg/l. In addition, Cd^2+^, Cr^6+^, and Zn^2+^ inhibited DBP degradation by LV-1 at all considered concentrations, but low concentrations of Pb^2+^, Cu^2+^, and Mn^2+^ enhanced DBP degradation. The main intermediates (mono-ethyl phthalate [MEP], mono-butyl phthalate [MBP], and phthalic acid [PA]) were identified in the DBP degradation process, thus a new biochemical pathway of DBP degradation is proposed. Furthermore, LV-1 also degraded other phthalates with shorter ester chains (DMP, DEP, and PA).

## 1. Introduction

Phthalate esters (PAEs) are a group of refractory organic compounds that are widely-used in coatings, medical product packaging, plastics, cosmetics, and building materials [1–2]. PAEs can easily migrate into the environment from plastic products and other related products because PAEs are not chemically bound to the polymeric matrix [3]. Numerous studies have revealed that PAEs and their metabolites can impact reproduction and behavior of mammals, even at very low concentrations [4–5]. Therefore, the environmental protection agency of many countries has classified most PAEs as top priority pollutants [3,6].

Di-*n*-butyl phthalate (DBP), which belongs to the class of PAEs, has been detected in various environments, such as soils, sediments, water, air, landfill leachates, plants, gas, and indoor dust [7–9]. In addition, many crops can absorb and store DBP, which leads to DBP entry into the food chain and threatens the health of mammals [6]. Therefore, it is critically important to efficiently remove DBP from the environment.

Indeed, it has been shown that several natural processes can remove DBP from the environment, including photolysis, hydrolysis, and biodegradation [10–11]. Due to the low rate of hydrolysis and photolysis, metabolic breakdown of DBP by microorganisms is regarded as a major process in the environmental degradation of DBP [12–13]. Many DBP-degrading bacterial strains have been isolated from various environmental samples [7,14–15], including approximately 25 genera, such as *Pseudomonas fluorescens* [16], *Rhodococcus* spp. [17], *Sphingomonas* spp. [18], and *Bacillus subtilis* [19]. However, discussions about biodegradation of DBP by bacterial consortia are limited [20]. It has been shown that high diversity can enhance survival of a bacterial consortium in different environments, and increase the biodegradation efficiency of organic pollutions [20–21]. Therefore, investigating the biodegradation of DBP by bacterial consortia is warranted.

In the present study a DBP-degrading bacterial consortium (LV-1) was enriched from river sludge, and the DBP degradation potential of LV-1 was investigated. The influence of pH, temperature, inoculum size, and heavy metal ions on DBP degradation by LV-1 was examined. GC-MS and Illumina sequencing technology were used to analyze the degradation intermediates of DBP and the bacterial community structure.

## 2. Materials and methods

### 2.1 Chemicals

Di-*n*-butyl phthalate (DBP; 99% purity) used in this research was purchased from Aladdin-reagent Co. (Shanghai, China). Methanol (HPLC grade), ethyl acetate (analytical grade), and other chemical reagents (analytical grade) were purchased from the Chinese Medicine Group (Shanghai, China).

### 2.2 Media and enrichment of the bacterial consortium

The bacterial consortium was enriched from river sludge collected from Kaifeng, Henan Province, China (114°35’E, 34°79’N). The river sludge was contaminated with domestic waste and sewage seriously over a long period of time. The medium used in the experiments was minimum salt medium (MSM), as previously described by Wu et al. [22].

The enrichment procedure for the DBP-degrading bacteria consortium was similar to our previous report [22]. 5g of river sludge were added to a 250-ml Erlenmeyer flask containing 100 ml of MSM and DBP (50 mg/l). The resulting suspension was cultured at 30°C and 175 rpm in the dark for 7 days. Then, 1 ml of the enrichment culture was serially transferred to fresh MSM containing 100, 200, 300, 400, 500, 1000, 1000, and 1000 mg/l of DBP (each enrichment step lasted 7 days). The final enrichment culture was designated as LV-1, and used for Illumina sequencing and further degradation experiments.

### 2.3 DNA extraction, amplification, and Illumina sequencing

Total genomic DNA of LV-1 was extracted using an EZ-10 Spin Column Genomic DNA Minipreps Kit (Bio Basic Inc., Markham, Ontario, Canada) following the protocol provided by the manufacturer. The primers and adapter sequences used for amplification of the V4 hypervariable region of the 16S rRNA gene were described by Caporaso et al. [23]. The PCR reactions and data processing were performed according to the description of Xiao et al. [24].

### 2.4 DBP degradation experiments using the bacterial consortium

LV-1 was grown in MSM with DBP (500 mg/l) as the sole source of carbon and energy, harvested after 48 h, thrice-washed with 0.05 mol/l of potassium phosphate buffer (pH 7.5). The washed cells were re-suspended in the same phosphate buffer (OD_600_ = 1.0) for application in the following degradation experiments.

Various amounts of DBP dissolved in methanol were added to 50-ml Erlenmeyer flasks, then incubated in a water bath rocker at 60°C to evaporate the methanol. Then, 1 ml of cell suspension and 19 ml of MSM were added to the Erlenmeyer flasks. The influence of the following environmental factors on DBP degradation were investigated within 48 h of incubation in MSM containing 500 mg/l of DBP at 175 rpm: temperature (15, 20, 25, 30, 35, 40, and 45°C); pH (4.0, 5.0, 6.0, 7.0, 8.0, 9.0, and 10.0); inoculum size (1.25, 2.5, 3.75, 5.00, 6.25, 7.5, 8.75, and 10.0%); Cd^2+^(2, 5, 10, 15, and 20 mg/l); Cr^6+^(10, 20, 30, 40, and 50 mg/l); and Pb^2+^, Mn^2+^, Zn^2+^, and Cu^2+^(50, 100, 200, 300, and 400 mg/l). When one parameter changed, other parameters are set as the commonly used condition in degrading of organic pollutant (pH 7.0, temperature 30°C, inoculum size 5%, and 175 rpm). After 48-h incubation, samples were withdrawn from the rocker platform, and the residual DBP in Erlenmeyer flasks was detected by ultra-performance liquid chromatography (UPLC). All experiments were performed in triplicate.

### 2.5 Effect of the initial DBP concentration on DBP biodegradation

Flasks containing cells suspension of LV-1 and DBP (initial concentration: 50, 100, 200, 300, 400, 500, and 1000 mg/l) were removed from the rocking platform (30°C and 175 rpm) at 12-h intervals. The samples were stored at 4°C for further UPLC analysis.

### 2.6 Analysis of DBP degradation intermediates

To analyze the biodegradation intermediates of DBP, samples were concentrated approximately 10-fold before gas chromatography–mass spectrometry (GC–MS; Agilent, USA) analysis. The detection procedure and operating conditions for GC-MS were according to He et al. [20].

### 2.7 Growth on other aromatic compounds

LV-1 was inoculated in MSM medium supplemented with other aromatic compounds (200 mg/l), including phthalic acid (PA), dimethyl phthalate (DMP), diethyl phthalate (DEP), dioctyl phthalate (DOP), and bisphenol A (BPA), to assess the potential ability to degrade these compounds. Cell growth was monitored turbidometrically at 600 nm after 48-h incubation. All experiments and controls were performed in triplicate.

### 2.8 Analytical methods

Extraction of residual DBP and degradation intermediates from the liquid was following the procedure described in our previous report [13]. The chromatographic conditions for detecting DBP by UPLC were as follows: mobile phase, methanol: water (90:10 [v/v]); the flow rate (0.5 ml/min); and UV wave-length (254 nm).

### 2.9 Data analysis

The influence of heavy metals on DBP degradation was statistically analyzed by SPSS 13.0, and the significant tests were conducted with ANOVA and multiple comparison.

## 3. Results and discussion

### 3.1 Enrichment and community analysis of LV-1

**Fig 1:**
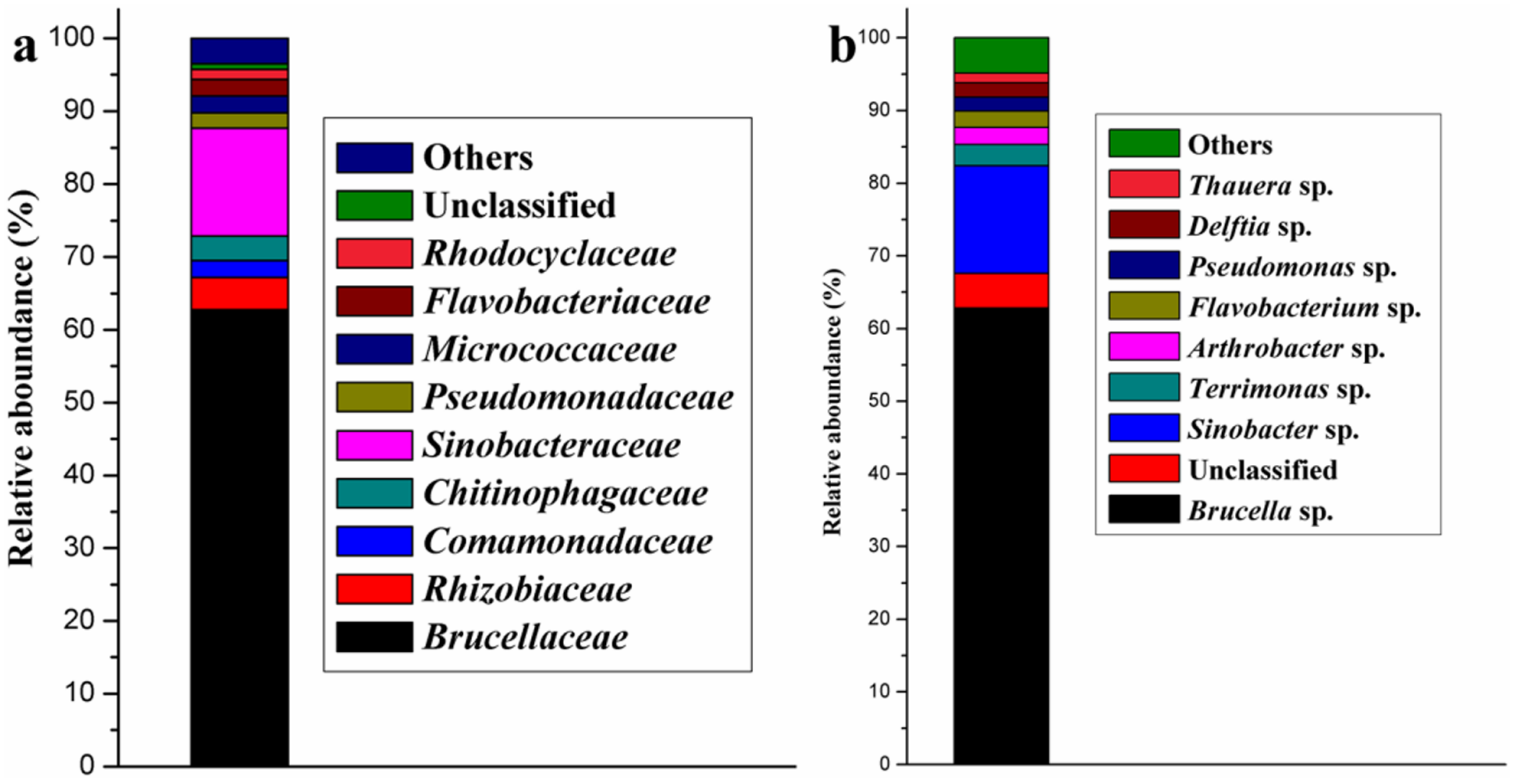
Bacterial community structure of LV-1. (a) at family level; (b) at genus level.

After eight transfers of the enrichment culture to fresh medium, the bacterial consortium (LV-1) was obtained. Community analysis revealed that LV-1 consisted of approximately 33 families; the predominant families were *Brucellaceae* (62.78%) and *Sinobacteraceae* (14.83%), and the sum of the two families accounted for 77% of all reads (Fig 1a). In addition, the relative abundance of *Rhizobiaceae*, *Comamonadaceae*, *Chitinophagaceae*, *Pseudomonadaceae*, *Micrococcaceae*, *Flavobacteriaceae*, and *Rhodocyclaceae* were 4.42%, 2.28%, 3.37%, 2.15%, 2.32%, 2.26%, and 1.36%, respectively.

At the genus level, LV-1 consisted of 48 genera, and the predominant genera were *Brucella* spp. (62.78%) and *Sinobacter* spp. (14.83%; Fig 1b). The relative abundance of *Terrimonas* spp., *Arthrobacter* spp., *Flavobacterium* spp., *Pseudomonas* spp., *Delftia* spp., and *Thauera* spp. were 2.93%, 2.32%, 2.26%, 1.95%, 1.95%, and 1.36%, respectively. Few reports have been published on degradation of organic pollutions by *Sinobacter* spp. and *Terrimonas* spp., and *Brucella* spp. has only been found in degrading oil and phenol [25–26]. Other genera detected in LV-1 were ubiquitous and have been reported many times in biodegradation of multiple organic pollutants in different environmental conditions, such as *Delftia* spp. [27], *Pseudomonas* spp. [16], *Arthrobacter* spp. [5], and *Flavobacterium* spp. [7], but the relative abundance of these genera was not high in LV-1. Besides, *Brucella* spp. is known to contain several pathogenic species, which can infect human and domestic animals. Therefore, the remediated area should be thoroughly disinfected after remediation of organic pollutants, which can reduce potential risk of pathogenic bacteria to human.

He et al. [20] reported a DBP-degrading bacterial consortium (HD-1), which was mainly composed of *Gordoni*a spp., *Achromobacter* spp., and *Burkholderia* spp. There are approximately 48 genera in the bacterial consortium of LV-1, among which 8 genera have a relative abundance >1%. High diversity of the bacterial consortium can enhance the microorganism survival ratio in different environments [20,28]. Bacterial consortium is more suitable for bioremediation than pure bacterial strains, suggesting that LV-1 is a suitable bacterial consortium in the bioremediation of DBP contamination.

### 3.2 Effects of temperature and pH on DBP degradation

**Fig 2:**
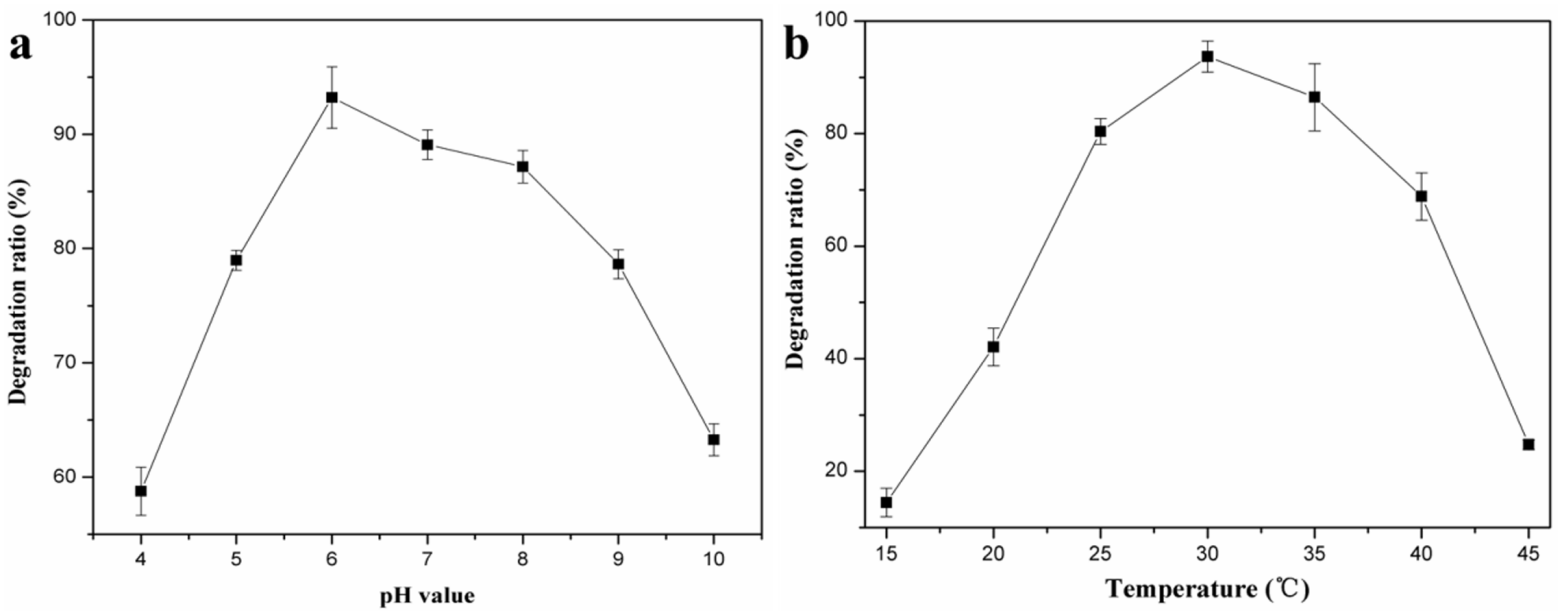
Effects of pH (a) and temperature (b) on degradation of DBP by LV-1.

Bacterial growth is pH- and temperature-sensitive. The effect of pH on DBP degradation by LV-1 is shown in Fig 2(a). The degradation ratio increased rapidly from 58.75% to 93.22% when the initial pH of the medium increased from 4.0 to 6.0. When the pH exceeded 6.0, the degradation ratio decreased slowly, indicating that the optimal pH for LV-1 degrading DBP was 6.0. In addition, the degradation ratio could be maintained at 89.07% and 87.16% when the initial pH increased to 7.0 and 8.0, respectively. DBP degradation ratio was > 58.75% in all considered initial pH values, indicating that LV-1 has a broad pH value in degrading DBP.

The influence of temperature on degradation of DBP is shown in Fig 2(b). The DBP degradation ratio increased from 14.43% to 93.68% as the temperature increased from 15°C to 30°C. However, the DBP degradation ratio decreased when the temperatures was higher than 30°C, indicating that the optimum temperature was 30°C. The degradation ratio could be maintained > 42.07% when the temperature is between 20°C to 40°C, indicating that LV-1 also has a relatively broad temperature in the degradation of DBP.

In previous reports, bacteria can degrade DBP at certain temperature or pH with a high degradation efficiency, such as 30°C [5], 35°C [3], pH 7.0 [29], pH 9.0 [5] etc. But the degradation efficiency decrease rapidly with the changes of pH and temperature. However, LV-1 can degrade DBP efficiently at a broad range of temperature and pH, which may directly relate to the high diversity of LV-1. LV-1 includes about 48 genera of bacteria, which made LV-1 more easily to adapt to different environments and degrade DBP with relatively higher efficiency.

### 3.3 Effect of inoculum size on DBP degradation

**Fig 3:**
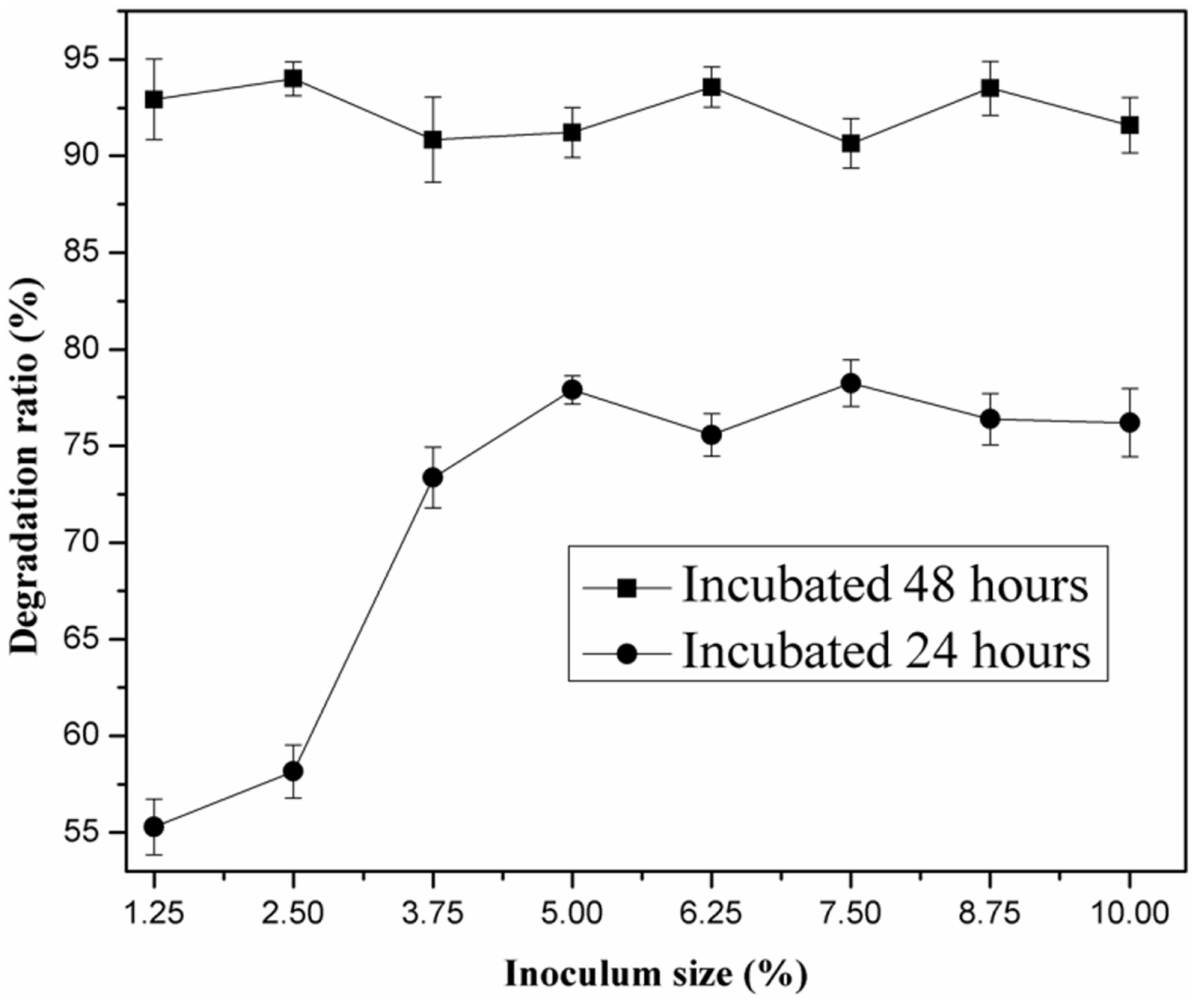
Effect of inoculum size on biodegradation of DBP by LV-1.

The influence of inoculum size on the DBP degradation ratio is shown in Fig 3. Within 24 h of incubation, the DBP degradation ratio was greatly influenced by the inoculum size. Nearly no DBP was degraded in the control experiments (the inoculum size at 0%). The degradation ratio just reached 55.28% with the inoculum size at 1.25%, and the degradation ratio increased quickly with the increase of the inoculum size. When the inoculum size was higher than 3.75%, the degradation ratio increased to approximately 73.5%, but the change in the degradation ratio of DBP was not apparent. When the incubation time increased to 48 h, the influence of inoculum size on the DBP degradation ratio was not significant, which indicating that the inoculum size has little influence on DBP degradation ratio within an incubation time ≥ 48 h. Wolski et al. [30] indicated that cultures inoculated with the lowest size had the highest substrate consumption rate, indicating that bacterial strains or enrichment can degrade the organic pollutant, even at a lower inoculum size. However, the bacterial strains or enrichments which are able to degrade organic pollutants under laboratory conditions may fail to show the same degradation ratio in a natural environment [31], and a higher inoculum size is still needed in the practical bioremediation process. Combined with the results of previous researches [2,5,22], an inoculum size of 5% was used in the following experiments.

### 3.4 Effect of heavy metal ions on DBP degradation

**Fig 4:**
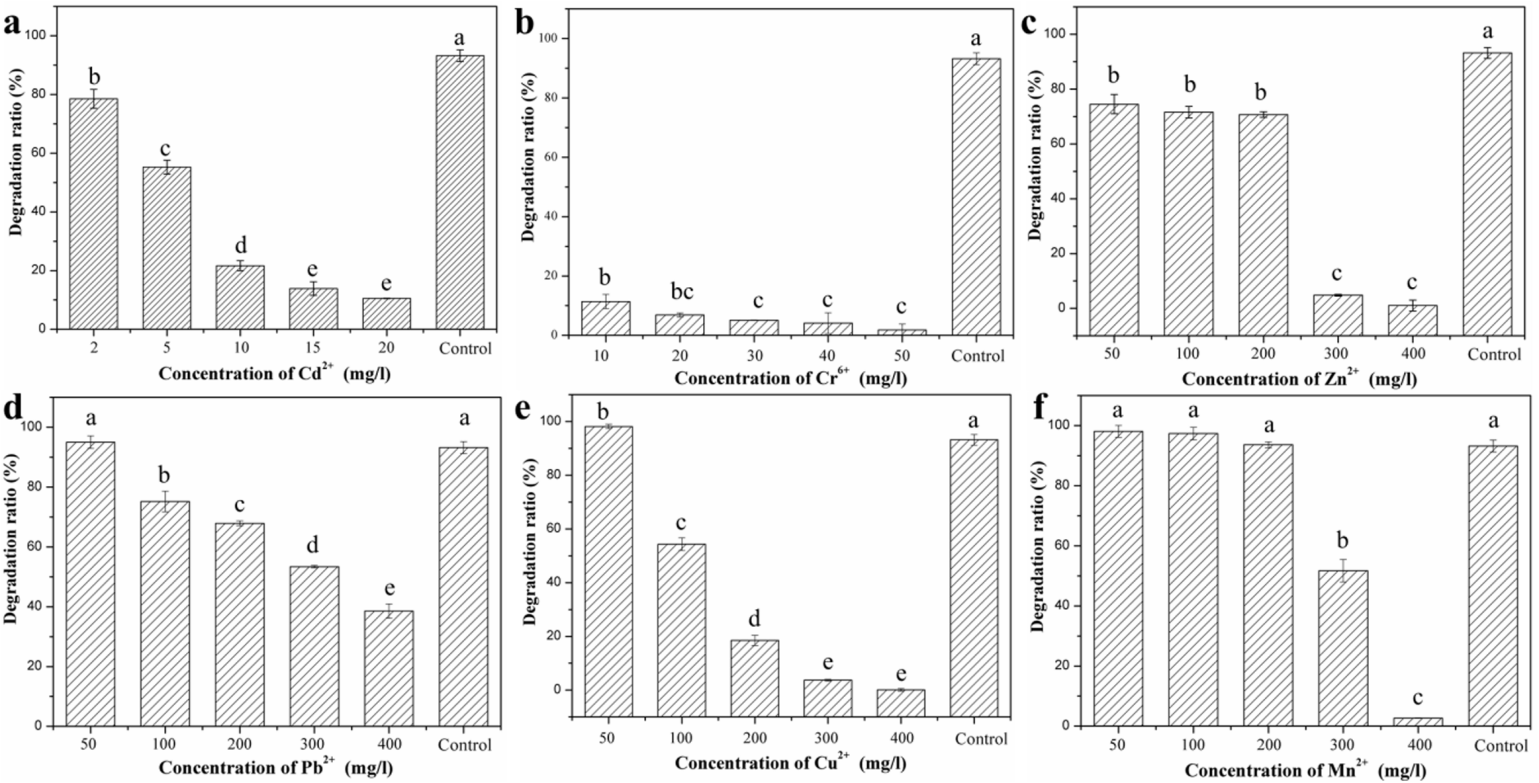
Effect of various concentrations of heavy metal ions on biodegradation of DBP by LV-1. (a) Cd^2+^; (b) Cr^6+^; (c) Zn^2+^; (d) Pb^2+^; (e) Cu^2+^; (f) Mn^2+^. The difference lowercases above the column indicate the influence of heavy metals on DBP degradation with significant differences.

The effect of heavy metal ions on degradation of DBP by LV-1 was investigated in detail. ANOVA revealed that all heavy metals tested in this research could influence biodegradation of DBP by LV-1 significantly (S1 Table). Multiple comparisons indicated that the influence of heavy metal on DBP degradation was concentration-depended (Table in S1 Table and Fig 4). Cd^2+^, Cr^6+^, and Zn^2+^ greatly inhibited the degradation activity of LV-1 at all considered concentrations. The DBP degradation ratio was 78.51% when the concentration of Cd^2+^ was 2 mg/l (Fig 4(a)). The degradation ratio decreased quickly with increase of the Cd^2+^ concentration, which decreased to 10.53% when the concentration of Cd^2+^ increased to 20 mg/l. LV-1 was more sensitive to Cr^6+^, and the degradation ratio ware only 22.40% and 1.82% when the concentration of Cr^6+^ was 10 and 50 mg/l (Fig 4(b)), respectively. The effect of Zn^2+^ on the degradation ratio was not serious when the concentration was < 200 mg/l (Fig 4(c)), and the degradation ratio was > 72.58%. However, the degradation ratio decreased to 4.82% and 1.08% when the concentration of Zn^2+^ increased to 300 and 400 mg/l, respectively.

Low concentrations of Pb^2+^, Cu^2+^, and Mn^2+^ enhanced biodegradation of DBP by LV-1. The degradation ratio reached 95.04% and 98.17%, respectively, when the concentration of Pb^2+^ and Cu^2+^ was 50 mg/l, which was higher than the value in control experiments (Fig 4(d) and 4(e)). The degradation ratio decreased quickly with an increase in the concentrations of Pb^2+^ and Cu^2+^, and the degradation of DBP was completely inhibited when the concentration of Cu^2+^ was 400 mg/l. Mn^2+^ enhanced the biodegradation of DBP by LV-1 when the concentration was < 200 mg/l (Fig 4(f)), but the degradation was inhibited when the concentration of Mn^2+^ was ≥ 300 mg/l. In fact, previous reports have shown that Mn^2+^ is an activator of different enzymes and can enhance the enzymatic reactions, such as protease [32], β-xylosidase [33], α-amylase [34], and leucine-rich repeat kinase 2 [35]. MnCl_2_ was also added in the MSM (0.0015 mg/l), but the concentration was too low for LV-1 to degrade DBP, indicating that the DBP degradation efficiency by LV-1 can be further improved through optimization of the composition of MSM.

Many researchers have reported the influences of heavy metals on bacteria growth [36–38]. Mrvčić et al. [37] found that the addition of Zn and Mn have no effect on the bacteria growth, while copper ion was highly toxic. Admas and Ghiorse [39]reported that Mn^2+^ have various effects on the growth of *Leptohrix discophora* strain SS-1 in batch cultures depending on the concentration added to the medium. Ravikumar et al. [40] found all identified bacterial species in their experiment are sensitive to Hg and Zn. In fact, the influence of heavy metals on the growth of bacteria is mainly affected by its influence on enzymes activity. Yu and Cheng [41] found that urease activity firstly increased with the addition of Cu, Pb and Cd, then showed declined trends. Zhang et al [42] reported that U has negative effects on all kinds of soil enzymes in their experiments, but Mn can promote the activity of peroxidase, and different concentration of Mn has different influence on the activities of sucrose. Besides, Pb also promoted the activity of peroxidase when its concentration is low. In our research, Pb^2+^, Cu^2+^ and Mn^2+^ promoted the growth of LV-1 at low concentration, whereas they inhibited the growth of LV-1 when their concentration increased. Cd^2+^, Cr^6+^ and Zn^2+^ inhibited the growth of LV-1 even at low concentration. That is to say, the influence of heavy metals on LV-1 is concentration-dependent, which is consistent well with previous researches [37,39,41–42].

### 3.5 DBP degradation characteristics with various initial concentrations

**Fig 5:**
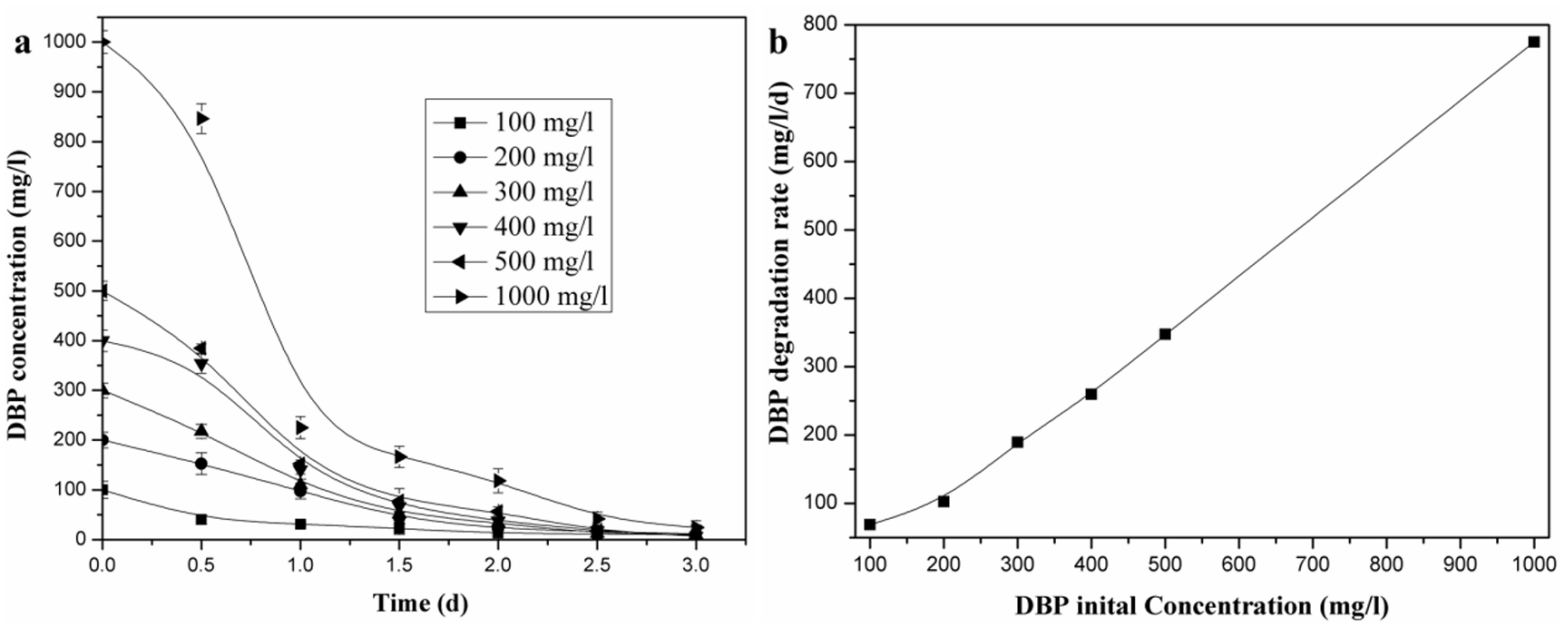
DBP degradation profiles of different DBP initial concentrations. (a) DBP degradation; (b) DBP degradation rate at first day.

The influence of the initial DBP concentration on DBP degradation by LV-1 was evaluated and the results are presented in Fig 5(a). DBP was degraded to non-detectable levels within 3 days when the initial concentration of DBP was < 500 mg/l; the degradation ratio reached 97.6%, even when the initial DBP concentration increased to 1000 mg/l. Although DBP was degraded at all considered initial concentrations, DBP degradation rates were clearly different within the first day. Fig 5(b) shows the degradation rate of DBP by LV-1 within the first day. The degradation rate of DBP by LV-1 increased from 69.0 to 775.0 mg/l/d with the initial concentration increasing from 100 to 1000 mg/l, indicating that a higher initial DBP concentration appeared to have no toxic inhibitory effect on the degradation ability of LV-1. Dou et al. [43] have also reported that degradation rate increased with an increase in the initial concentration. Fang et al. [18] reported biodegradation of DEP by *Sphingomonas* spp. and the degradation rate was also initially concentration-dependent. Our results are consistent with existing researches [18,43]. Kinetic analysis revealed that the degradation of DBP by LV-1 was best fitted by first-order kinetics when the initial concentration was < 300 mg/l, and the half-life was 0.636 (100 mg/l) and 0.705 (200 mg/l) days, respectively.

### 3.6 Analysis of DBP degradation intermediates

**Fig 6:**
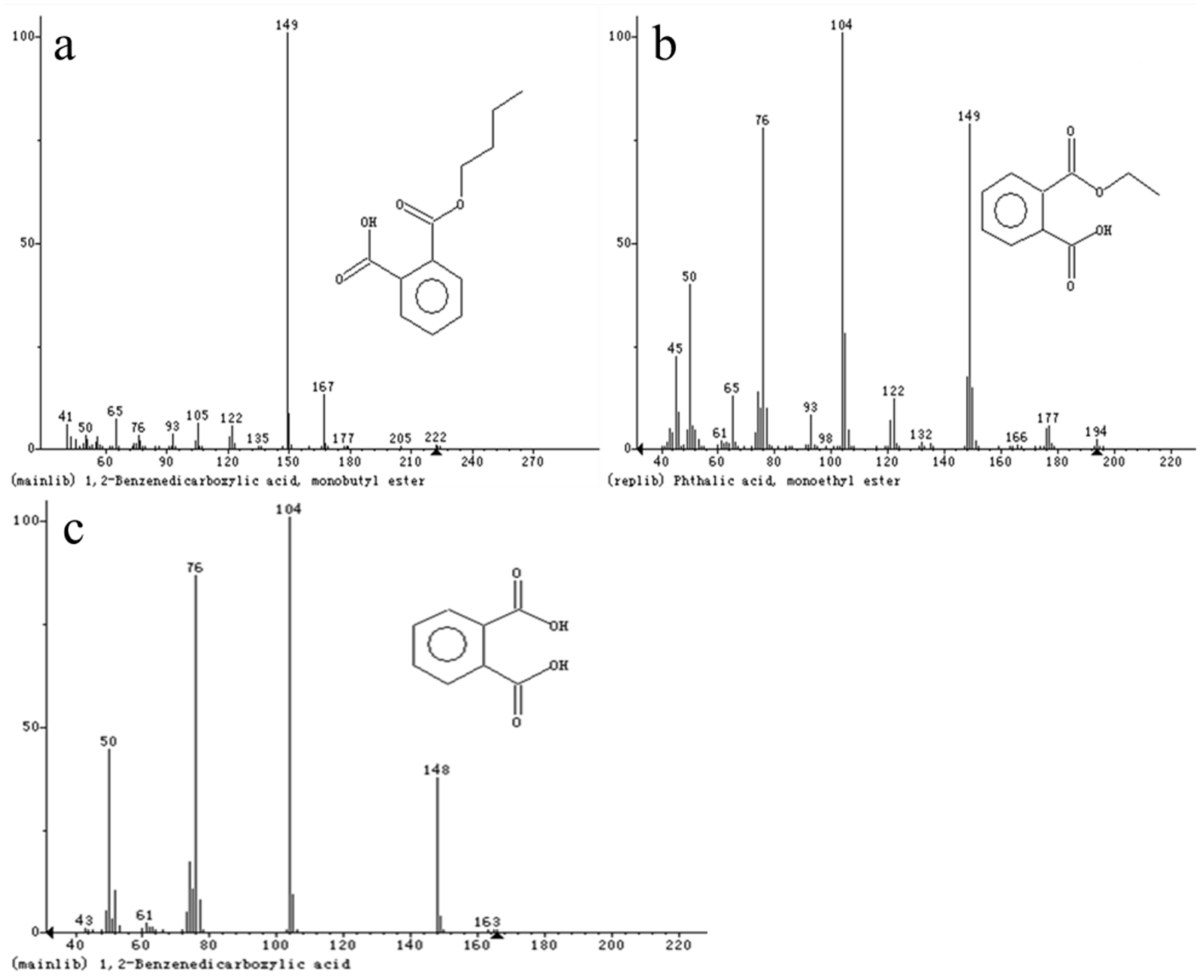
MS spectra of the intermediates of DBP by LV-1. (a) MBP, (b) MEP, and (c) PA.

To explore the biochemical degradation pathways of DBP by LV-1, the degradation intermediates of DBP were monitored by GC-MS at 8, 16, 24, 32, 40, and 48 h. Three major intermediates (mono-butyl phthalate [MBP], mono-ethyl phthalate [MEP], and phthalic acid [PA]) were identified from these samples (Fig 6). The existence of the MEP suggests that the biodegradation of DBP by LV-1 might be β-oxidation alternating with ester hydrolysis. DBP can remove one ethyl group by β-oxidation to form butyl ethyl phthalate (BEP) [44], and the BEP can be transformed to MBP through ester hydrolysis, to then form MEP and PA via the next round of β-oxidation and ester hydrolysis (First possibility). The metabolite of BEP was not detected in this study, possibly because small amounts of BEP accumulated and the concentration of BEP was lower than the detection limits. Besides, this still has another possibility. The first side-chain was entirely removed by ester hydrolysis and formed MBP. After that, the next side-chain can be removed by β-oxidation and ester hydrolysis to form PA. And BEP is non-existent in this degradation pathway (Second possibility).

**Fig 7:**
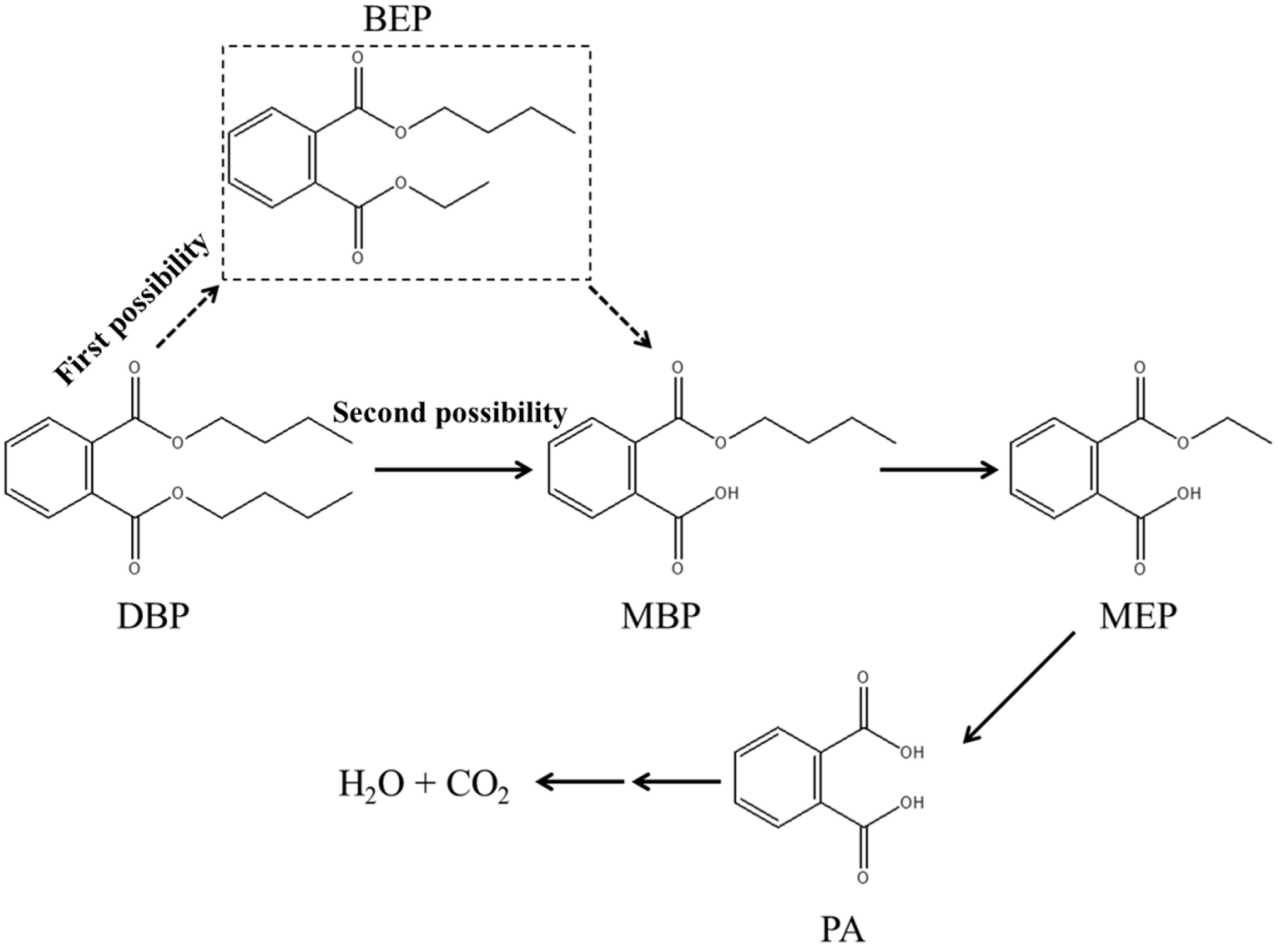
Proposed biochemical degradation pathway for DBP by LV-1. The dashed box indicates the inferred intermediate that was not detected in this study.

Based on the above results, the tentative metabolic pathway for degradation of DBP by LV-1 is proposed (Fig 7). The degradative pathway (First possibility) is different from most previous reports [13,45]. Previous reports indicated that only PA could be detected in the DBP degradation process with a bacterial consortium [11,20]. [46]also reported that intermediate products were only detected when the organic pollutants were degraded by pure cultures, and no intermediate products were detected when the organic pollutants were degraded by mixed cultures or bacterial consortia. The community composition of LV-1 may be the main reason for these results observed in this study. The total relative abundance of *Brucella* spp. (62.78%) and *Sinobacter* spp. was about 78% of LV-1, but few of them have been reported in degrading organic pollutant. Therefore, the enriched *Brucella* spp. and *Sinobacter* spp. might partially explain why the degradative pathway of LV-1 is different with previous reports. Besides, [22] reported that bacteria can degrade DOP through β-oxidation, ester hydrolysis, and trans-esterification, which are consistent well with our results. But the second possible degradative pathway is similar with most previous reports [7,13,45].

### 3.7 Substrate utilization

MSM supplemented with different organic pollutants (each 200 mg/l) was used to determine the range of substrate utilization by LV-1. The results (Table 1) showed that phthalates with shorter ester chains were readily degraded by LV-1 (DMP, DEP, DBP, and PA), whereas those with longer ester chains were only marginally degraded (DOP), which is consistent with our previous report [2].

**Table 1:** Growth profile of LV-1 on various of organic pollutions.

	Substrates						
	Control	PA	DMP	DEP	DBP	DOP	BPA
OD_600_	0	0.133±0.008	0.159±0.002	0.128±0.007	0.191±0.008	0.021±0.009	0.017±0.002

## 4. Conclusion

In summary, a bacterial consortium was enriched from river sludge and the bacterial community structure was analyzed. *Sinobacter* spp. and *Terrimonas* spp. were reported to degrade DBP for the first time. The influence of pH, temperature, initial substrate concentration, and heavy metals on degradation of DBP by LV-1 was analyzed. The intermediates of DBP biodegradation were analyzed by GC-MS, and the degradation pathway is proposed.

## Supporting information

S1 Table Variance analyze of the influence of heavy metal ions on biodegradation of DBP by LV-1. (a) Cd^2+^; (b) Cr^6+^; (c) Zn^2+^; (d) Pb^2+^; (e) Cu^2+^; (f) Mn^2+^.
(DOCX)
