# Supplementary material for: Biodegradation of di-n-butyl phthalate by bacterial consortium LV-1 enriched from river sludge
Source: PLoS One. 2017 May 25;12(5):e0178213. doi: 10.1371/journal.pone.0178213 (PMC5444784; doi:10.1371/journal.pone.0178213)
Supplement: S1 Table — (a) Cd2+; (b) Cr6+; (c) Zn2+; (d) Pb2+; (e) Cu2+; (f) Mn2+. (DOCX) [file pone.0178213.s001.docx]

Variance analyze of the influence of heavy metal ions on biodegradation of DBP by LV-1. (a) Cd^2+^; (b) Cr^6+^; (c) Zn^2+^; (d) Pb^2+^; (e) Cu^2+^; (f) Mn^2+^

(a) Cd^2+^

1. Variance analysis

|  | Df | Sum Sq | Mean Sq | F value | Pr(>F) | Significant |
| --- | --- | --- | --- | --- | --- | --- |
| Concentration | 5 | 1.8766 | 0.3753 | 798.8 | 1.05e-14 | *** |
| Residuals | 12 | 0.0056 | 0.0005 |  |  |  |

Significant codes: 0.001 ‘***’; 0.01 ‘**’; 0.05 ‘*’;

2. Significant differences between different concentrations

| Cd^2+^ Concentration | means | Mark |
| --- | --- | --- |
| 0 | 0.9322 | a |
| 2 | 0.7851 | b |
| 5 | 0.5525 | c |
| 10 | 0.2164 | d |
| 15 | 0.1386 | e |
| 20 | 0.1053 | e |

(b) Cr^6+^

1. Variance analysis

|  | Df | Sum Sq | Mean Sq | F value | Pr(>F) | Significant |
| --- | --- | --- | --- | --- | --- | --- |
| Concentration | 5 | 1.9238 | 0.3848 | 871 | 6.29e-15 | *** |
| Residuals | 12 | 0.0053 | 0.0004 |  |  |  |

Significant codes: 0.001 ‘***’; 0.01 ‘**’; 0.05 ‘*’;

2. Significant differences between different concentrations

| Concentration | means | Mark |
| --- | --- | --- |
| 0 | 0.9322 | a |
| 10 | 0.11395 | b |
| 20 | 0.06845 | bc |
| 30 | 0.05095 | c |
| 40 | 0.041 | c |
| 50 | 0.0182 | c |

(c) Zn^2+^

1. Variance analysis

|  | Df | Sum Sq | Mean Sq | F value | Pr(>F) | Significant |
| --- | --- | --- | --- | --- | --- | --- |
| Concentration | 5 | 2.3272 | 0.4654 | 1084 | 1.7e-15 | *** |
| Residuals | 12 | 0.0052 | 0.0004 |  |  |  |

Significant codes: 0.001 ‘***’; 0.01 ‘**’; 0.05 ‘*’;

2. Significant differences between different concentrations

| Concentration | means | Mark |
| --- | --- | --- |
| 0 | 0.9322 | a |
| 50 | 0.74523 | b |
| 100 | 0.71585 | b |
| 200 | 0.7073 | b |
| 300 | 0.0482 | c |
| 400 | 0.01075 | c |

(d) Pb^2+^

1. Variance analysis

|  | Df | Sum Sq | Mean Sq | F value | Pr(>F) | Significant |
| --- | --- | --- | --- | --- | --- | --- |
| Concentration | 5 | 0.7384 | 0.14768 | 325.4 | 2.24e-12 | *** |
| Residuals | 12 | 0.0054 | 0.00045 |  |  |  |

Significant codes: 0.001 ‘***’; 0.01 ‘**’; 0.05 ‘*’;

2. Significant differences between different concentrations

| concentration | means | Mark |
| --- | --- | --- |
| 0 | 0.9322 | a |
| 50 | 0.9504 | a |
| 100 | 0.752 | b |
| 200 | 0.67895 | c |
| 300 | 0.53395 | d |
| 400 | 0.3854 | e |

(e) Cu^2+^

1. Variance analysis

|  | Df | Sum Sq | Mean Sq | F value | Pr(>F) | Significant |
| --- | --- | --- | --- | --- | --- | --- |
| Concentration | 5 | 2.8748 | 0.5750 | 2404 | 2e-16 | *** |
| Residuals | 12 | 0.0029 | 0.0002 |  |  |  |

Significant codes: 0.001 ‘***’; 0.01 ‘**’; 0.05 ‘*’;

2. Significant differences between different concentrations

| Concentration | means | Mark |
| --- | --- | --- |
| 0 | 0.9322 | a |
| 50 | 0.98165 | b |
| 100 | 0.54355 | c |
| 200 | 0.18485 | d |
| 300 | 0.03715 | e |
| 400 | 0.01 | e |

(f) Mn^2+^

1. Variance analysis

|  | Df | Sum Sq | Mean Sq | F value | Pr(>F) | Significant |
| --- | --- | --- | --- | --- | --- | --- |
| Concentration | 5 | 2.2377 | 0.4475 | 989.9 | 2.93e-15 | *** |
| Residuals | 12 | 0.0054 | 0.0005 |  |  |  |

Significant codes: 0.001 ‘***’; 0.01 ‘**’; 0.05 ‘*’;

2. Significant differences between different concentrations

| Concentration | means | Mark |
| --- | --- | --- |
| 0 | 0.9322 | a |
| 50 | 0.981 | a |
| 100 | 0.9737 | a |
| 200 | 0.93665 | a |
| 300 | 0.51715 | b |
| 400 | 0.0267 | c |
